# Interstitial Lung Disease in Dermatomyositis Without Myositis-Specific and Myositis-Associated Autoantibodies: Study of a Series of 72 Patients From a Single Cohort

**DOI:** 10.3389/fimmu.2022.879266

**Published:** 2022-05-06

**Authors:** Fang Chen, Jinping Wang, Puli Zhang, Yu Zuo, Lifang Ye, Guochun Wang, Xiaoming Shu

**Affiliations:** Department of Rheumatology, Key Laboratory of Myositis; Beijing Key Laboratory for Immune Mediated Inflammatory Diseases, China-Japan Friendship Hospital, Beijing, China

**Keywords:** negative myositis autoantibody, interstitial lung disease, RPILD, dermatomyositis, myositis specific autoantibody, myositis associated autoantibody

## Abstract

**Objective:**

The clinical features of interstitial lung disease (ILD) in patients with dermatomyositis (DM) and negative myositis autoantibodies had not been exactly demonstrated previously. This study aimed to describe and expand the phenotype of interstitial lung disease (ILD) in this cohort of patients.

**Methods:**

A total of 1125 consecutive Chinese patients with idiopathic inflammatory myopathies (IIM) between 2006 and 2020 were screened retrospectively. All proven cases of isolated ILD with both negative myositis-specific autoantibodies (MSA) and negative myositis-associated autoantibodies (MAA) were selected for inclusion. The clinical features and outcome among this group, MDA5^+^DM (DM patients with positive anti-MDA5 antibody) and ASS (patients with positive anti-aminoacyl tRNA synthetases antibodies were recorded and compared.

**Results:**

Of 1125 IIM patients with an average follow-up of 6 years, 154 DM patients with negative MSA and MAA (MSA/MAA) were identified, with an ILD incidence of 46.8%. DM-ILD Patients with negative MSA/MAA presented younger age at onset (p<0.001), lower incidence of elevated CA153 (p=0.03) and fever (p=0.04)than those ILD patients with MDA5^+^DM and ASS.The estimated high-resolution computed tomography patterns of ILD showed non-specific interstitial pneumonia (66.6%), followed by organizing pneumonia in patients with negative MSA/MAA. OP pattern was more common in patients with MDA5^+^DM (69.7%), and the ratios of the OP (48.7%) and NSIP (51.3%) patterns were almost equal in patients with ASS. Of these DM-ILD patients with negative MSA/MAA, 25% developed rapidly progressive interstitial lung disease (RP-ILD). Patients with RP-ILD had a shorter disease duration (p=0.002), higher percentage of positive ANA(p=0.01) and organizing pneumonia patterns (p=0.04), elevated CYFRA211(p=0.04) and decreased FiO2/PaO2 (p<0.001) than those with chronic progressive ILD. The incidence of OP pattern in RP-ILD patients with negative MSA/MAA was lower than in those RPILD patients with MDA5^+^ DM (75%) and ASS (89%) (p=0.006). The cumulative 5- and 10-year survival rates in the DM-ILD patients with negative MSA/MAA were 91% and 88%, respectively, during the long-term follow-up study. And they had more favorable survival rate compared with ILD patients with MDA5^+^DM and ASS (p<0.001). An independent prognostic factor was identified as decreased PaO2/FiO2 (hazard ratio, 0.97; p=0.004].

**Conclusions:**

This study indicates DM-ILD patients with negative MSA/MAA had favorable long-term outcomes. Decreased baseline PaO2/FiO2 acted as an independent prognostic factor for this group of patients.

## Introduction

Idiopathic inflammatory myopathy (IIM) is a heterogeneous autoimmune disease characterized by muscle weakness and multiple extramuscular manifestations, including varying degrees of skin, joint and lung involvement. Interstitial lung disease (ILD) is the most crucial extramuscular manifestation of IIM because of its high prevalence and mortality rate (1). Poor survival and impaired quality of life are most commonly observed in patients with acute or chronic progressive ILD in patients with IIM. Serum myositis-specific autoantibodies (MSAs) and negative myositis-associated autoantibodies (MAAs) are identified in recent years. Many of these are associated with a unique clinical subset of IIM, making them useful for predicting and monitoring certain clinical manifestations (2, 3). The association between ILD and these antibodies has been confirmed in several studies. Anti-melanoma differentiation-associated gene 5(MDA5) antibodies with amyopathic dermatomyositis (ADM) or clinical amyopathic dermatomyositis (CADM) are often complicated by rapidly progressive ILD (RP-ILD) (4). Studies have confirmed an association between anti-aminoacyl tRNA synthetases (ARS) antibodies and IIM-ILD (5). The anti-Ro-52 antibody is one of MAAs, which also associated with ILD and RP-ILD in previous studies and our team’s studies (6, 7). However, little attention has been paid to the special group of dermatomyositis with ILD, in which both MSA and MAA are negative. The serological, radiological, and clinical outcomes of these patients are unclear. This study aimed to describe a cohort of ILD patients with dermatomyositis with negative MSA and MAA(MSA/MAA).

## Materials and Methods

### Study Design and Subjects

This was a retrospective cohort study of adult patients who visited the Department of Rheumatology, China-Japan Friendship Hospital, from January 2006 to July 2020. Muscle biopsies were performed for all patients. Patients with a definite diagnosis of DM fulfilled the Bohan and Peter classification criteria and the 239th ENMC International Classification of Dermatomyositis (8). All patients provided written informed consent in accordance with the Declaration of Helsinki. This study was approved by the China-Japan Friendship Hospital review board (approval number: 2016-117). Baseline demographic data, clinical data, laboratory data and radiographic data at presentation were extracted from the medical records.

The presence of ILD was determined by high-resolution computed tomography (HRCT) findings. RP-ILD was defined as a condition of worsening radiological interstitial change with progressive dyspnea and hypoxemia within 1 month of the onset of respiratory symptoms. chronic progressive ILD(CP-ILD) was defined as an asymptomatic, nonrapidly progressive ILD or slowly progressive ILD over 3 months (9). HRCT scan patterns were classified as non-specific interstitial pneumonia (NSIP), usual interstitial pneumonia (UIP), and organizing pneumonia (OP) by two experienced radiologists, according to the 2013 American Thoracic Society (ATS) and European Respiratory Society (ERS) policies (10).

### Detection of Serum Autoantibodies

To avoid confounding and variability associated with testing techniques and reference values between laboratories, we only included patients who had MSA and MAA autoantibody profiles performed at the first visit at the Rheumatology Laboratory of China-Japan Friendship Hospital. In patients included before 2016, MSA status was assessed retrospectively based on stored baseline serum samples using the Euroline assay. Since seroconversion was observed, repeated MSA measurements during follow-up were performed in all enrolled patients, and serum aliquots were frozen at −80°C until assayed. Autoantibodies (antigen including MDA5,PL-12,PL-7,Jo-1,EJ,OJ,KS,Mi-2,TIF1-**γ**,NXP2,SRP,SAE,PM-Scl-75,PM-Scl-100,Ku,Ro-52,HMGCR) were measured by immunoblotting(Euroimmun, Germany; MBL, Japan). Anti-nuclear and ENA antibodies were detected using commercial kits from Euroimmun Germany. Each sample was analyzed in triplicate to ensure accuracy.

### Inclusion and Exclusion Criteria

The inclusion criteria were as follows: (1) eligible patients with DM who had complete medical records and follow-up information. (2) Both MSA- and MAA-negative DM patients with ILD during the initial visit and follow-up time.

The exclusion criteria were as follows: (1) DM without ILD, (2) any MSA- or MAA-positive DM with ILD, (3) patients with other connective tissue diseases, and (4) Patients with evidence of pulmonary infections at their first admission, which were identified by clinical diagnosis and pathogens.

### Statistical Analysis

The chi-squared or Fisher’s exact test was used for binary data. Student’s t-test or Mann–Whitney U-test or One-way ANOVA analysis were used for continuous data. Survival probability was evaluated using the Kaplan-Meier method, and survival curves were compared using the log-rank test. To demonstrate possible significant explanatory risk factors for death, we analyzed prognostic factors using univariate and multivariate Cox regression models, and the hazard ratios (HRs) and 95% confidence intervals for death were calculated using Cox proportional-hazards models; two-sided p<0.05, considered statistically significant. All statistical calculations were performed using the SPSS software (SPSS, Chicago, IL, USA).

## Results

### Screening ILD Patients of DM With Negative MSA/MAA

**Figure 1:**
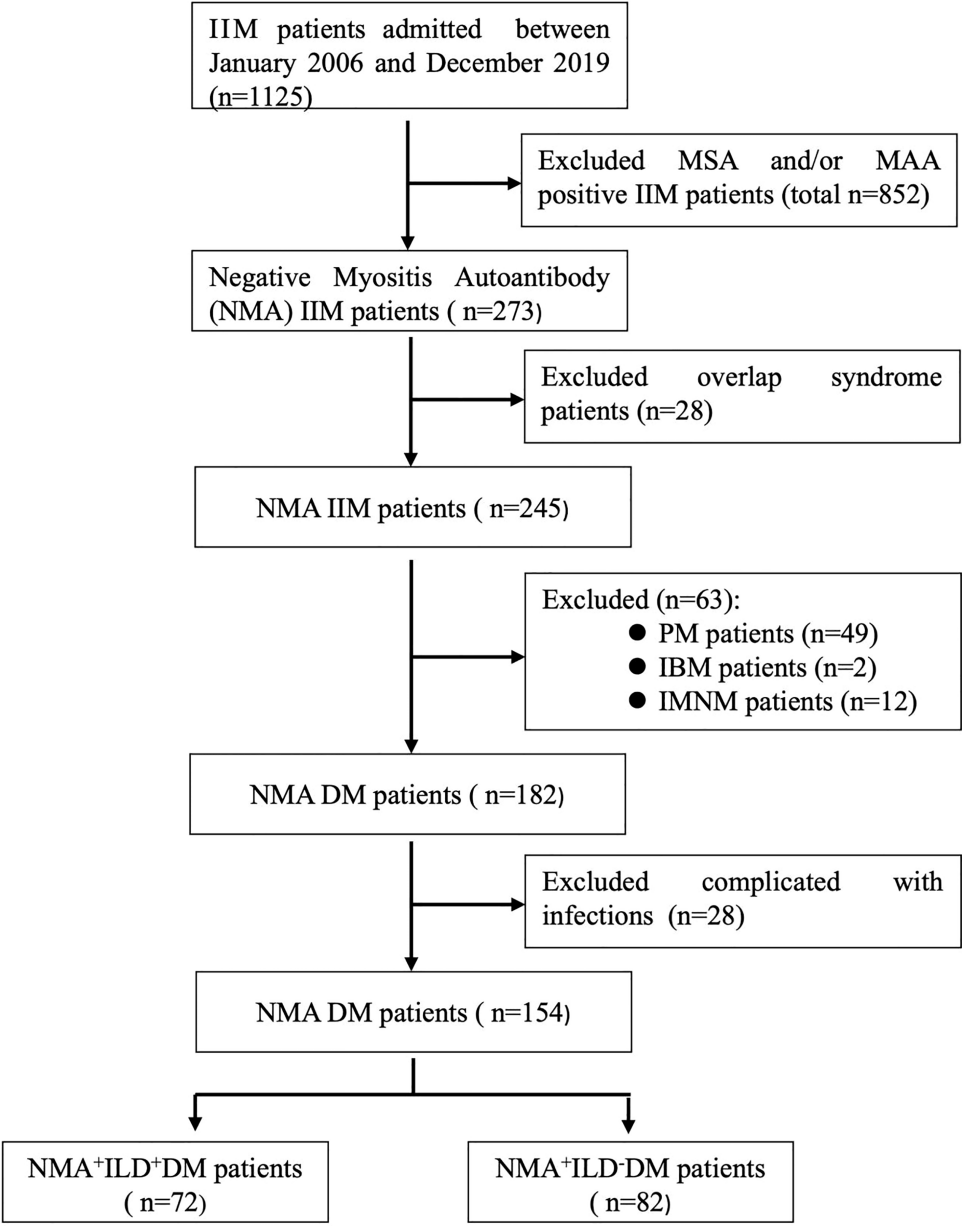
Flowchart of DM-ILD patients with negative MSA/MAA. IIM, idiopathic inflammatory myopathy; MSA, myositis specific autoantibody; MAA, myositis associated autoantibody; PM, polymyositis; IBM, inclusion body myopathy; IMNM, immune mediated necrotizing myopathy; ILD, interstitial lung disease.

The study cohort included 1,125 patients with IIM. A total of 273 patients tested negative for both MSA and MAA. 28 patients with other connective tissue diseases were excluded. A total of 182 (74%) of 245 patients with IIM were diagnosed as DM. To avoid potential interference from pulmonary infection, 28 patients were excluded because of complications of pulmonary infection at their first visit to our hospital. A total of 154 patients with DM and negative MSA/MAA were enrolled, of them,72 patients (46.8%) were complicated by ILD (**Figure 1**). The characteristics of all the DM patients with negative MSA/MAA were mainly include: lymphocytic infiltrates and MHC-I expression in all patients, perifascicular atrophy observed in 55.6% of patients (n=86).

### Clinical Features of Negative MSA/MAA Associated ILD in DM Patients

The general and clinical characteristics of the patients are shown in **Table 1** and **Supplemental Table S1**. The prevalence of ILD in DM patients with negative MSA/MAA was 46.7%; however, ILD was much less common in patients with Juvenile dermatomyositis (JDM) (1.4%) (p=0.03). ILD Patients with negative MSA/MAA showed older age at onset (p<0.001), shorter disease duration (p=0.007), and elevated levels of serum ferritin (p=0.008) and C-reactive protein (p=0.016) compared with patients without ILD. Importantly, the incidence of Gottron’s sign (p=0.049), mechanic’s hands (p<0.001), and arthralgia (p=0.002) was higher in DM-ILD patients with negative MSA/MAA. In addition, ILD patients with negative MSA/MAA had higher rates of tumor markers CEA and CA153 (both p=0.003) (**Supplememtal Table S1**).

**Table 1:** The comparison of clinical features among ILD patients with negative MSA/MAA, anti MDA5 and anti-ARS antibodies.

ILD	DM with negative MSA/MAA (n=72)	MDA5^+^DM (n=175)	ASS (n=158)	P value
Age at onset (years)	47.1 ± 12	49 ± 11	54 ± 11.8	<0.001
Gender (F,%)	48 (66.7%)	117 (66.8%)	112 (70.8%)	0.68
Disease duration (weeks)	4.5 (2.3,13.5)	3 (2,6)	8 (2,24)	<0.001
Heliotrope sign (n,%)	42 (58.3%)	132 (75.4%)	34 (21.5%)	<0.001
mechanic’s hand (n,%)	37 (51.4%)	84/91 (48%)	77/81 (48.7%)	0.8
Gottron’s sign (n,%)	49 (68%)	141 (81.7%)	57 (36.1%)	<0.001
muscle weakness (n,%)	48 (66.7%)	100 (57%)	77 (48.7%)	0.03
Arthralgia (n,%)	34 (47.2%)	95/80 (54.3%)	80 (50.6%)	0.57
Fever (n,%)	23 (31.9%)	86 (49.1%)	72 (45.6%)	0.04
Perlungual erythematous (n,%)	8 (11.1%)	27 (15.4%)	6 (3.8%)	0.002
Lymphocyte counts (cell/ul)	1110 (600,1581)	750 (510,1060)	1000 (1440,2057)	<0.001
CK (IU/L)	79 (42,287)	51 (28,107)	114 (51,605)	0.48
LDH (IU/L)	252 (204,367)	287 (225,378)	291 (204,396)	1
Ferritin (ng/ml)	244 (123,669)	542 (210,1378)	188 (79,375)	0.01
CRP (mg/dl)	0.53 (0.23,0.96)	0.61 (0,1)	1 (0.2,2.3)	0.1
ESR (mm/h)	17 (7,36)	22 (13.42)	21 (11,43)	0.2
Elevated CEA (n,%)	14 (24.1%)	81 (50.9%)	22 (17.2%)	<0.001
Elevated CA7-24 (n,%)	8 (30.7%)	30 (28.8%)	9 (16.4%)	0.18
Elevated CA125 (n,%)	11 (19%)	4 (2.5%)	28 (21.9%)	<0.001
Elevated CA199 (n,%)	10 (17.5%)	28 (17.6%)	29 (22.7%)	0.5
Elevated CA153 (n,%)	12 (21%)	60 (38%)	52 (40.3%)	0.03
Elevated NSE (n,%)	12 (41.4%)	51 (48%)	23 (30.7%)	0.001
Elevated CYFRA21-1 (n,%)	18 (56.3%)	78 (72%)	63 (73.2%)	0.16
FVC%	87.8 ± 25	82.5 ± 23	75 ± 19.8	0.075
DLco%	66 ± 17	65 ± 17.6	63.6 ± 16	0.6
HRCT patterns				
OP	23 (31.9%)	122 (69.7%)	77 (48.7%)	<0.001
NSIP	48 (66.7%)	53 (30.3)	81 (52.6%)	
UIP	1 (1.4%)	0	0	

(Table 1 Breakdown): Continuous data were presented as M (mean) ± SEM (standard error of the mean), or medians (interquartile range). Binary data were presented as n (%) of the patients. DM, dermatomyositis; CK, creatine kinase; LDH, Lactate Dehydrogenase; FVC, Forced Vital Capacity; DLco, diffusing capacity for carbon monoxide; UIP, usual interstitial pneumonia; NSIP, nonspecific interstitial pneumonia; OP, organizing pneumonia.

The comparison of clinical features of ILD were made among DM with negative MSA/MAA,MDA5^+^DM (DM patients with positive anti-MDA5 antibody) (n=175) and ASS (patients with positive anti-aminoacyl tRNA synthetases antibodies (n=158)patients in our cohort **(** **Table 1** **)**. DM Patients with negative MSA/MAA presented younger age at onset (p<0.001), lower incidence of elevated CA153 (p=0.03) and fever (p=0.04)than those ILD patients with MDA5^+^DM and ASS. Patients with negative MSA/MAA and ASS showed higher lymphocyte counts (p<0.001), lower levels of ferritin (p=0.01), lower incidence of elevated CEA (p<0.001) and higher incidence of elevated CA125 (p<0.001) than those in MDA5^+^ DM Patients. The duration in ASS^+^ILD patients were longer than those in the other two groups of patients (p<0.001).

### Characteristics of the Estimated HRCT Patterns in DM-ILD Patients With Negative MSA/MAA

It had previously not exactly indicated the estimated high-resolution computed tomography (HRCT) patterns in DM-ILD patients with negative MSA/MAA. We next evaluated the HRCT patterns in this group of patients. The results showed that the non-specific interstitial pneumonia (NSIP) pattern was the most frequent finding (66.6%), followed by the OP patterns (32.8%), and the UIP patterns (0.6%) in DM-ILD patients with negative MSA/MAA. The HRCT patterns were different in patients with negative MSA/MAA from patients with MDA5^+^DM and ASS (p<0.001). OP pattern was more common in patients with anti-MDA5 antibodies (69.7%), and the ratios of the OP (48.7%) and NSIP (51.3%) patterns were almost equal in patients with anti-ARS antibodies **(** **Table 1** **)**.

Considering that DM-ILD patients with negative MSA/MAA had a higher prevalence of mechanic’s hand and arthritis/arthralgia, we wanted to further explore whether there were associations between HRCT patterns and the clinical characteristics of mechanic’s hand and arthritis/arthralgia. As shown in **Supplementary Table S2**, no significant differences were noted between negative MSA/MAA associated ILD patients with mechanic hands and arthralgia/arthritis (both p>0.05). However, patients with mechanic’s hand showed more common OP than those with arthralgia/arthritis (34.2% vs. 22.9%, p=0.38), while patients with arthralgia/arthritis were more common with NSIP than those with mechanic’s hand (76.5% vs. 63.2%, p=0.206) (**Supplementary Table S2**).

### Prevalence and Characteristics of RP-ILD in DM Patients With Negative MSA/MAA

Our previous study indicated that RP-ILD is mainly found in patients with anti-MDA5 and anti-ARS or isolated anti-Ro-52 positive DM (7, 11, 12). Notably, 18 patients (25%) presented with RP-ILD, suggesting that patients with DM-ILD patients with negative MSA/MAA could develop RP-ILD. Most patients presented with chronic progressive ILD (CP-ILD) (75%) (**Table 2**).

**Table 2:** Prevalence and characteristics of RP-ILD in DM-ILD patients with negative MSA/MAA patients.

	RP-ILD (N=18)	CP-ILD (N=54)	P value
DM type			0.5
DM (n,%)	15 (83.3%)	48 (88.9%)	
CADM (n,%)	3 (16.6%)	5 (9.3%)	
JDM (n,%)	0 (0%)	1 (1.8%)	
Age at onset (years)	49 ± 13	46 ± 12	0.8
Gender (F,%)	12 (66.7%)	36 (66.7%)	1
Disease duration (weeks)	3 (1,4)	7 (3,19.5)	0.002
Heliotrope sign (n,%)	11 (61.1%)	31 (57.4%)	0.78
Mechanic’s hand (n,%)	10 (55.6%)	27 (50%)	0.68
Gottron’s signs (n,%)	12 (66.7%)	37 (68.5%)	0.88
Muscle weakness (n,%)	10 (55.6%)	38 (70.4%)	0.25
Arthralgia (n,%)	6 (33.3%)	29 (53.7%)	0.13
Fever (n,%)	8 (44.4%)	15 (27.8%)	0.18
ANA positive (n,%)	11 (68.8%)	17 (34%)	0.01
T cells counts (cell/ul)	926 ± 512	962 ± 600	0.9
CK (IU/L)	78 (39,294)	79 (43,298)	0.87
LDH (IU/L)	283 (235,537)	237 (196,360)	0.11
Ferritin (ng/ml)	311 (176,1034)	244 (88,651)	0.17
Elevated CEA (n,%)	4 (30.7%)	10 (22.2%)	0.53
Elevated CA7-24 (n,%)	3 (50%)	6 (28.6%)	0.33
Elevated CA125 (n,%)	2 (15.4%)	9 (20%)	0.7
Elevated CA199 (n,%)	2 (15.4%)	8 (18.2%)	0.8
Elevated CA153 (n,%)	5 (38.5%)	7 (15.9%)	0.09
Elevated NSE (n,%)	4 (50%)	8 (38.1%)	0.68
Elevated CYFRA21-1 (n,%)	8 (88.9%)	10 (43.4%)	0.04
CRP (mg/dl)	0.96 (0.39,3.22)	0.45 (0.21,0.76)	0.007
ESR (mm/h)	16.5 (6,41)	17 (7.5,35.5)	0.92
PaO2/FiO2	300 ± 54	423 ± 49	<0.001
FVC%	69 ± 18.9	92 ± 27	0.007
FEV1%	67 ± 19.8	87 ± 27	0.022
DLco%	48 ± 11	66 ± 18	0.003
HRCT patterns			
OP	10 (55.6%)	13 (24.1%)	0.04
NSIP	8 (44.4%)	40 (74.1%)	
UIP	0 (%)	1 (1.8%)	

(Table 2 Breakdown): Continuous data were presented as M (mean) ± SEM (standard error of the mean), or medians (interquartile range). Binary data were presented as n (%) of the patients. DM, dermatomyositis; CADM, Clinical amyopathic dermatomyositis; JDM, Juvenile dermatomyositis; CK, creatine kinase; LDH, Lactate Dehydrogenase; FVC, Forced Vital Capacity; FEV1, Forced Expiratory Volume In 1s; DLco, diffusing capacity for carbon monoxide; UIP, usual interstitial pneumonia; NSIP, nonspecific interstitial pneumonia; OP, organizing pneumonia.

Furthermore, we compared the clinical features of the RP-ILD and CP-ILD groups in the DM Patients with negative MSA/MAA group. The clinical features of the two groups are summarized in **Table 2**. Disease duration was significantly shorter in the RP-ILD group than that in the CP-ILD group (p=0.002). The ratio of ANA positivity in the RP-ILD group was higher than that in the CP-ILD group (P=0.01). More importantly, PaO2/FiO2, FVC%, FEV1%, and DLco% were all decreased in the RP-ILD group compared to the CP-ILD group (all p<0.01). In addition, RP-ILD patients presented a higher ratio of elevated CYFRA21-1 levels, more than twice as high as those of CP-ILD patients (88.9% vs. 43.5%, p=0.04). Notably, although OP and NSIP were the main HRCT patterns in the two groups, OP containing consolidations in the lower lung zone of HRCT findings was more common in the RP-ILD group (55.6% vs. 24%, p=0.01). No significant differences were found in skin rash, arthralgia, muscle weakness, serum CK, LDH, lymphocytes, serum ferritin, ESR, and CRP levels between the two groups (all p>0.05).

There were 110 ILD patients with MDA5^+^ DM (62.9%) and 70 patients with ASS^+^ILD (44.3%) developed RP-ILD in our cohort. The clinical features of RP-ILD among DM patients with negative MSA/MAA, MDA5^+^ DM and ASS patients were also compared. RPILD patients with negative MSA/MAA and ASS showed higher incidence of elevated CA125 (p<0.001) and positive ANA (p=0.01), lower incidence of elevated CEA (p<0.001), higher lymphocyte counts (p<0.001) and lower levels of ferritin (p=0.007) than those patients with MDA5^+^DM. The incidence of OP pattern in RPILD patients with negative MSA/MAA was lower than in those patients with MDA5^+^ DM (75%) and patients with ASS (89%) (p=0.006) **(** **Table 3** **)**.

**Table 3:** The comparison of clinical features among RPILD patients with negative MSA/MAA, anti-MDA5 and anti-ARS antibodies.

RPILD	DM with negative MSA/MAA (N=18)	MDA5+DM (N=110)	ASS (N=70)	P value
Age at onset (years)	49 ± 13	51.6 ± 10	54 ± 12	0.18
Gender (F,%)	12 (66.7%)	75/35	55/15	0.28
Disease duration (weeks)	3 (1,4)	3 (2,6)	5 (1.5,36)	0.02
Heliotrope sign (n,%)	11 (61.1%)	81 (73.6%)	13 (18.6%)	<0.001
mechanic’s hand (n,%)	10 (55.6%)	48/62 (43.6%)	35 (50%)	0.5
Gottron’s signs (n,%)	12 (66.7%)	89/21 (81%)	22 (31.4%)	<0.001
muscle weakness (n,%)	10 (55.6%)	62/48	27/43	0.06
Arthralgia (n,%)	6 (33.3%)	46	28	0.3
ANA positive (n,%)	11 (68.8%)	45/44 (50.6%)	48/18 (72.8%)	0.01
Lymphocyte counts (cell/ul)	1256 ± 657	770 ± 487	1598 ± 947	<0.001
CK (IU/L)	78 (39,294)	50 (28,117)	95 (50,424)	0.11
LDH (IU/L)	283 (235,537)	326 (255,404)	314 (225,430)	0.44
Ferritin (ng/ml)	311 (176,1034)	706 (360,1694)	175 (81,499)	0.007
Elevated CEA (n,%)	4 (30.7%)	60/39 (61%)	16 (27.1%)	<0.001
Elevated CA7-24 (n,%)	3 (50%)	16/50 (24%)	5 (18.5%)	0.26
Elevated CA125 (n,%)	2 (15.4%)	3 (3%)	18 (30.1%)	<0.001
Elevated CA199 (n,%)	2 (15.4%)	18 (18.3%)	17 (28.8%)	0.26
Elevated CA153 (n,%)	5 (38.5%)	48 (49.5%)	28 (47.5%)	0.75
Elevated NSE (n,%)	4 (50%)	37 (52%)	16 (42%)	0.6
Elevated CYFRA21-1 (n,%)	8 (88.9%)	57 (78%)	36 (82%)	0.7
CRP (mg/dl)	0.96 (0.39,3.22)	1 (0.2,1.7)	1 (0.2,3.8)	0.09
ESR (mm/h)	16.5 (6,41)	29 (17,51)	26 (12,43)	0.1
PaO2/FiO2	300 ± 54	308 ± 58	310 ± 46	0.8
FVC%	69 ± 18.9	67 ± 18.2	68 ± 17	0.8
DLco%	48 ± 11	46 ± 12	49 ± 12	0.9
HRCT patterns				
OP	10 (55.6%)	83 (75%)	62 (89%)	0.006
NSIP	8 (44.4%)	27 (24%)	8 (11%)	

(Table 3 Breakdown): Continuous data were presented as M (mean) ± SEM (standard error of the mean), or medians (interquartile range). Binary data were presented as n (%) of the patients. DM, dermatomyositis; CK, creatine kinase; LDH, Lactate Dehydrogenase; FVC, Forced Vital Capacity; DLco, diffusing capacity for carbon monoxide; UIP, usual interstitial pneumonia; NSIP, nonspecific interstitial pneumonia; OP, organizing pneumonia.

### Prognosis and Predictors of DM-ILD Patients With Negative MSA/MAA

**Figure 2:**
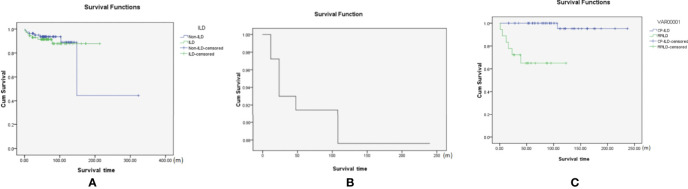
**(A)** The Kaplan-Meier survival analysis of negative MSA/MAA patients with or without ILD(p>0.05). **(B)** The cumulative 5 and 10-year survival rate of the DM-ILD patients with negative MSA/MAA was 91% and 88%; **(C)** Kaplan-Meier survival analysis of RP-ILD and CP-ILD patients with negative MSA/MAA (with p<0.05).

**Figure 3:**
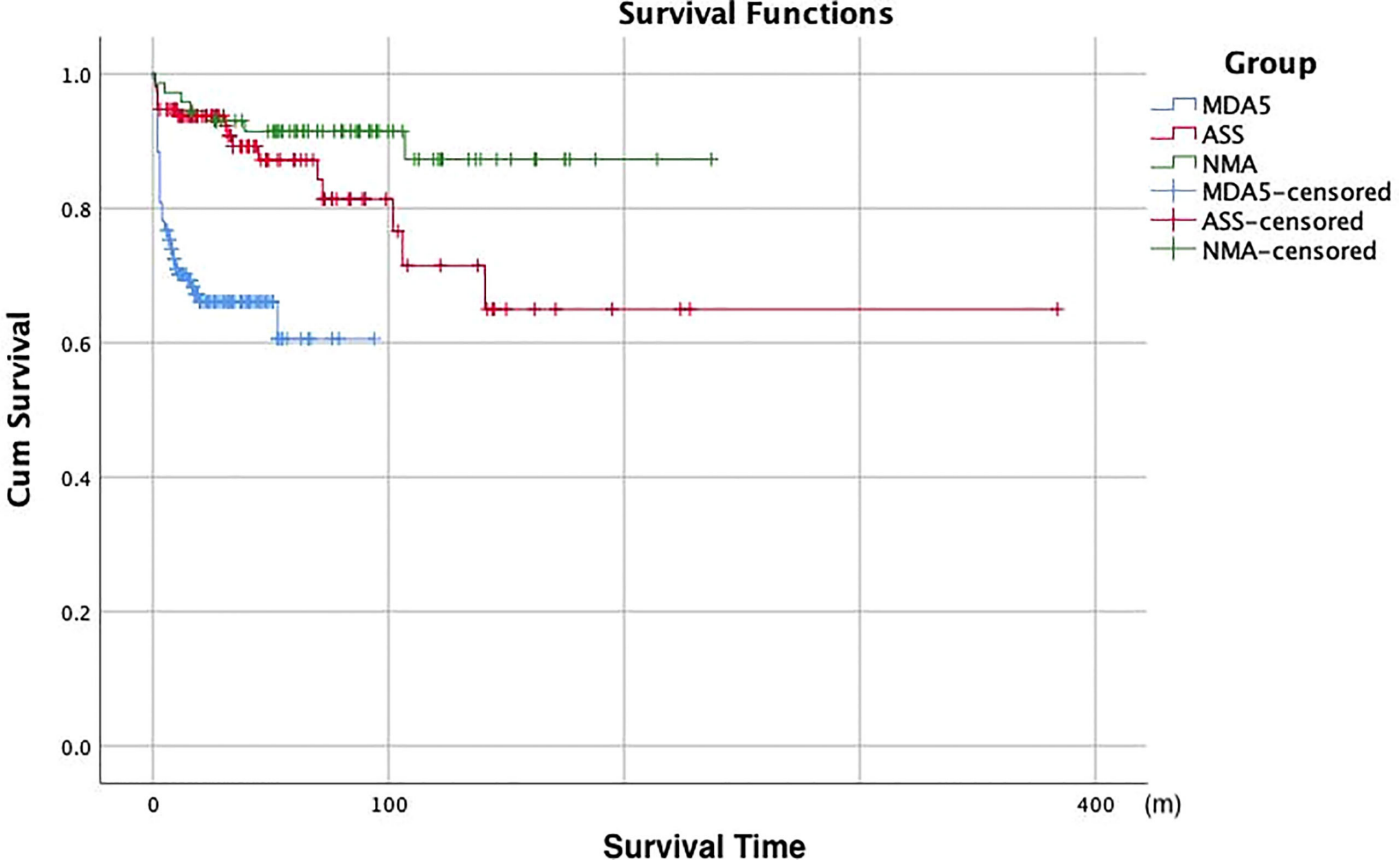
Kaplan-Meier survival analysis of ILD patients with negative MSA/MAA,MDA5^+^DM patients and ASS patients. NMA, negative myositis autoantibody.

We explored the prognosis of all DM patients with negative MSA/MAA (with and without ILD). After a median follow-up period of 6 years, there were both seven patients died in the patients with ILD and without ILD. All deaths occurred in 9.1% of the patients with negative MSA/MAA. In brief, among the seven patients without ILD, two of seven patients died of respiratory failure, one of seven patients died of severe asthma, and four of seven patients died of cancer (three lymphomas and one esophageal cancer). All seven patients in the ILD group died of respiratory failure. The Kaplan-Meier survival analysis showed no statistically significant difference in prognosis between sero-negative patients with ILD and those without ILD (P>0.05) (**Figure 2A**). **Figure 2B** also showed that the cumulative 5-and 10-year survival rates of DM-ILD patients with negative MSA/MAA were 91% and 88%, respectively. In addition, **Figure 2C** showed that the overall survival rate was lower in the RP-ILD group than in the CP-ILD group (p<0.05). Of the seven patients who died, two RP-ILD and one CP-ILD patients complicated by severe infection after glucocorticoids and immunosuppressive treatments. The Kaplan-Meier survival analysis were also conducted among patients with DM-ILD patients with negative MSA/MAA, MDA5^+^DM and ASS. the DM-ILD patients with negative MSA/MAA had the most favorable prognosis while MDA5^+^DM associated ILD patients had the worst prognosis (p<0.001) **(** **Figure 3** **)**. The cumulative 5 year survival rates of ILD patients with MDA5^+^ DM and ASS were 69% and 87% respectively.

To determine the predictors of DM-ILD patients with negative MSA/MAA, we first compared the differences in demographic characteristics of the surviving and dead NMA^+^ILD^+^DM groups during follow-up. As shown in **Table 4**, there were significant differences in disease duration, RP-ILD, elevated CYERA-21-1, ferritin decreased PaO2/FiO2, and DLCO% (p=0.009, <0.001, 0.02, 0.048,0.003, and 0.024, respectively). Next, we used univariate and multivariate analyses to compare the risk factors between the death and survival groups in patients with NMA^+^ILD^+^DM. Variables with P ≦ 0.05 were considered possible confounders and those previously reported risk factors, including serum ferritin, LDH, and decreased T cells, were also retained in subsequent multivariate Cox proportional hazard analysis. According to the univariate analysis, RP-ILD (HR=32, p=0.002), decreased PaO2/FiO2 (HR=0.978, p<0.001), and DLco% (HR=0.91, p=0.04) were predictors of prognosis for DM-ILD patients with negative MSA/MAA. Age- and sex-adjusted multivariate regression analysis revealed that only decreased PaO2/FiO2 (HR=0.97, p=0.013) was an independent predictor of prognosis in this group of patients (**Table 5**).

**Table 4:** Comparison of clinical characteristics between the dead and survival DM-ILD patients with negative MSA/MAA.

	Death (N=7)	survival (N=65)	P value
Age at onset (years)	48 ± 21	46.9 ± 11	0.29
Gender (F) (n,%)	5/2 (71.4%)	43/22 (66.2%)	1
Disease duration (M)	1 (1,3)	3 (6,16)	0.009
RP-ILD (n,%)	6 (85.7%)	12 (18.5%)	<0.001
Heliotrope sign (n,%)	4 (57%)	38 (58.5%)	1
Mechanic’s hand (n,%)	4 (57%)	33 (50.8%)	1
Gottron’s signs (n,%)	6 (85.7%)	38 (58.5%)	0.23
Muscle weakness (n,%)	5 (71.4%)	43 (66.2%)	1
Arthralgia (n,%)	4 (57%)	30 (46.2%)	0.7
Fever (n,%)	4 (57%)	19 (29.2%)	0.19
ANA positive (n,%) (N=65)	2 (33.3%)	25 (42.3%)	1
Decreased T cells counts	3 (50%)	17 (34.7%)	0.65
Elevated CK (n,%)	1 (14.3%)	22 (33.8%)	0.4
Elevated LDH (n,%)	4 (57%)	31 (49.2%)	1
Ferritin	552 (226,1153)	225 (106,649)	0.048
Elevated CRP (n,%) (N=67)	3 (42.9%)	19 (32.2%)	0.6
Elevated ESR (n,%) (N=67)	3 (42.9%)	29 (48.3%)	1
Elevated CEA (n,%) (N=58)	3 (42.9%)	11 (21.6%)	0.34
Elevated CA7-24 (n,%) (N=27)	2/2 (50%)	7 (30.4%)	0.58
Elevated CA125 (n,%) (N=58)	1 (14.3%)	10 (19.6%)	1
Elevated CA199 (n,%) (N=57)	1 (14.3%)	9 (18%)	1
Elevated CA153 (n,%) (N=57)	3 (42.9%)	9 (18%)	0.15
Elevated NSE (n,%) (N=32)	0 (0%)	12 (48%)	0.1
Elevated CYFRA21-1 (n,%) (N=32)	6 (100%)	12 (46.2%)	0.02
PaO2/FiO2	238 (310,343)	357 (429,448)	0.003
FVC%	71 ± 25	87 ± 27	0.21
FEV1%	68 ± 26	83 ± 27	0.25
DLco%	43 ± 8	62 ± 18	0.024
HRCT patterns OP (n, %) NSIP (n, %) UIP (n, %)	4 (57.1%) 3 (42.9%) 0 (%)	18 (27.7%) 46 (70.8%) 1 (1.5%)	0.22

(Table 4 Breakdown): Continuous data were presented as M (mean) ± SEM (standard error of the mean), or medians (interquartile range). Binary data were presented as n (%) of the patients; RPILD, rapidly progressive interstitial lung disease; CK, creatine kinase; LDH, Lactate Dehydrogenase; CRP, c reactive protein;ESR, erythrocyte sedimentation rate; FVC, Forced Vital Capacity; FEV1, Forced Expiratory Volume In 1s; DLco, diffusing capacity for carbon monoxide; UIP, usual interstitial pneumonia; NSIP, nonspecific interstitial pneumonia; OP, organizing pneumonia.

**Table 5:** Univariate and multivariate prognostic analyses of risk factors in DM-ILD patients with negative MSA/MAA.

	Univariate		Multivariate	
	HR (95%CI)	P value	HR (95%CI)	P value
Disease duration	–	0.27	–	–
RP-ILD	32(3.5,295)	0.002	–	–
Ferritin	–	0.38		
LDH	–	0.8		
Decreased T cells	–	0.9		
Elevated CYFRA21-1	–	0.25	–	–
PaO2/FiO2	0.978(0.967,0.99)	<0.001	0.97(0.95,0.99)	0.013
DLco%	0.91(0.84,0.99)	0.04		

(Table 5 Breakdown), RPILD, rapidly progressive interstitial lung disease; LDH, Lactate Dehydrogenase; DLco, diffusing capacity for carbon monoxide.

## Discussion

Many studies have found that anti-ARS, anti-MDA5, and anti-Ro-52 antibodies are strongly associated with PM/DM-ILD (4–7). However, a cohort of patients with DM, without these autoantibodies, is also complicated by ILD. The present study from a large single center illustrated the clinical and radiologic characteristics and outcomes of patients with DM complicated by ILD without MSAs and MAAs for the first time. Our study suggested that DM-ILD patients with negative MSA/MAA had unique clinical characteristics and HRCT patterns. The cohort had long-term favorable outcomes, although some patients could develop RP-ILD. Our study expands the phenotype of interstitial lung disease (ILD) in patients with DM.

The prevalence of ILD in the DM patients with negative MSA/MAA was 46.7%. The prevalence of ILD was 84.5% in MDA5-DM,87.3% in ASS-PM/DM, and 58% in patients with Ro-52-DM in our center (7, 11, 12). DM-ILD patients with negative MSA/MAA is associated with Gottron’s sign, mechanic’s hands, arthralgia, elevated levels of serum ferritin, CEA, and CA153. As mechanic’s hands and arthralgia usually exist in patients with ASS, it is unclear whether undetected anti-synthase antibodies exist in this group of patients. However, 76% of ILD patients with negative MSA/MAA had elevated ferritin levels, while only 36% of patients with ASS^+^ILD had elevated serum ferritin levels in our previous study (11). Besides, the age at onset,the disease duration and the incidence of Gottron’s sign and fever are differed in patients with negative MSA/MAA and ASS in the present study. Elevated serum tumor markers CEA and CA153 were found in DM-ILD patients with negative MSA/MAA as well as MDA5^+^DM and ASS. Recent research has indicated a positive correlation between serum tumor marker levels and CTD-ILD. Higher levels of CA153 and CYFRA21-1 suggest an increased risk of developing ILD (13). Our previous and other recent studies indicated that the tumor markers CEA, CA-199, and CYFRA21-1 were associated with ILD in DM (14–16). Our results also suggest that elevated levels of serum CEA and CA153 could be useful biomarkers for detecting ILD in DM patients with negative MSA/MAA in the clinical setting.

We also analyzed the estimated HRCT pattern of ILD patients with negative MSA/MAA. NSIP was the dominant pattern in this cohort (66.6%), whereas the OP pattern was more common in patients with anti-MDA5 antibodies, and the ratios of the OP and NSIP patterns were almost equal in patients with anti-ARS antibodies in our cohort. To note, Patients with DM-ILD patients with negative MSA/MAA presented younger age at onset and lower incidence of fever than those ILD patients with MDA5^+^DM and ASS in our cohort. The findings indicated that patients with DM-ILD patients with negative MSA/MAA had unique clinical features and estimated HRCT pattern.

RP-ILD was observed mainly in patients with anti-MDA5 antibody-positive DM and those with anti-ARS antibodies in previous studies (17–19). Among the ILD patients with negative MSA/MAA, 25% developed RP-ILD in our study. Therefore, it was necessary to be vigilant that some DM patients with negative MSA/MAA developed RP-ILD although the ratio of RP-ILD was lower in this group of patients than in the MDA5^+^DM and ASS patients. RP-ILD in patients with negative MSA/MAA was associated with positive ANA and elevated CYFRA21-1 levels. Of the patients with RP-ILD, 84.6% had positive ANA and 88.9% had elevated CYFRA211.which indicated positive ANA and elevated CYFRA21-1 levels may be useful markers for the development of RP-ILD; however, further prospective studies are needed to verify this. RPILD patients with negative MSA/MAA and ASS showed lower incidence of elevated CEA, higher lymphocyte counts and lower levels of ferritin than those RPILD patients with MDA5^+^ DM, which were consistent with previous studies (16).

Importantly, although NSIP was the main pattern in patients with ILD, OP containing consolidations in the lower lung zone of HRCT findings was more common in the RP-ILD group (55.6%). A similar association was found in RP-ILD patients of DM with anti-MDA5, anti-ARS, and anti-Ro-52 antibodies in our present and previous study (7, 16). This could remind clinicians that OP containing consolidations in the lower lung zone of HRCT may predict the development of RP-ILD in patients with DM, despite the presence or absence of myositis autoantibodies.

Long-term follow-up was conducted for all DM patients with negative MSA/MAA. There was no statistical difference in prognosis between the patients with and without ILD in negative MSA/MAA groups. The cumulative 5- and 10-year survival rates of patients with DM-ILD patients with negative MSA/MAA were 91% and 88%, respectively. And The Kaplan-Meier survival analysis showed the DM-ILD patients with negative MSA/MAA patients had the most favorable prognosis while MDA5^+^ DM^+^ILD had the worst prognosis, All of which indicating that DM-ILD patients with negative MSA/MAA had a favorable prognosis, although some patients may develop RP-ILD. Univariate analysis showed that RP-ILD, decreased PaO2/FiO2, and DLco% were risk prognostic factors, whereas multivariate regression analysis revealed that only decreased PaO2/FiO2 was an independent predictor of prognosis. There may be two reasons why RP-ILD was not an independent prognostic risk factor. One explanation is that RP-ILD patients responded well to intensive treatment with methylprednisolone in combination with CSA and/or intravenous CYC. Three of the seven patients died of complications of severe infections during treatment instead of death due to RP-ILD. Another explanation may be the small sample size for death, which may have biased the results of the prognostic analysis. Several studies have shown that ferritin, LDH, decreased lymphocytes, KL-6, FVC, and tumor markers are associated with poor prognosis in DM (14, 15, 20, 21). However, these risk factors were not associated with the prognosis of DM-ILD patients with negative MSA/MAA. These differences suggest that DM-ILD patients with negative MSA/MAA had an isolated clinical phenotype. Based on these findings, we are conducting ongoing research to identify new myositis autoantibodies and explore the disease mechanisms of DM-ILD patients with negative MSA/MAA.

The present study has several limitations as a retrospective study. First, we used immunoblotting kits to detect MSA and MAA. The specificity may be lower in comparison with the gold standard Immunoprecipitation. However, the immunoblotting kit is well proven and widely used in clinical practice. Second, there may be undescribed antibodies existed in the cohort, which need further identification. Finally, as mentioned previously, prognostic analysis may have biased results, as the number of deaths was small. More data should be collected to confirm our results in the future.

## Conclusion

NSIP existed in two-thirds of the estimated HRCT patterns in NMA^+^ILD^+^DM, while OP, especially containing consolidations in the lower lung zone, was the main pattern in patients with RP-ILD. Although 25% of ILD patients developed RP-ILD, the overall mortality was favorable in the NMA^+^ILD^+^DM group at long-term follow-up.

## Data Availability Statement

The original contributions presented in the study are included in the article/**Supplementary Material**. Further inquiries can be directed to the corresponding author.

## Ethics Statement

This study was approved by the China-Japan Friendship Hospital review board. Written informed consent to participate in this study was provided by the participants’ legal guardian/next of kin.

## Author Contributions

FC and XS conceived and designed the study. FC and JW collected clinical data. PZ conducted the follow-ups and recorded the outcomes. FC, YZ, and LY performed the autoantibody detection and statistical analysis. FC and XS wrote and edited the manuscript. XS and GW supervised the study. All authors contributed to the article and approved the submitted version.

## Funding

This study was funded by the Fundamental Research Funds for the Central Universities (No. 3332020074) and Elite Medical Professionals project of China-Japan Friendship Hospital(No.ZRJY2021-GG14).

## Conflict of Interest

The authors declare that the research was conducted in the absence of any commercial or financial relationships that could be construed as a potential conflict of interest.

## Publisher’s Note

All claims expressed in this article are solely those of the authors and do not necessarily represent those of their affiliated organizations, or those of the publisher, the editors and the reviewers. Any product that may be evaluated in this article, or claim that may be made by its manufacturer, is not guaranteed or endorsed by the publisher.
