# Supplementary material for: Interstitial Lung Disease in Dermatomyositis Without Myositis-Specific and Myositis-Associated Autoantibodies: Study of a Series of 72 Patients From a Single Cohort
Source: Front Immunol. 2022 May 6;13:879266. doi: 10.3389/fimmu.2022.879266 (PMC9120579; doi:10.3389/fimmu.2022.879266)
Supplement: Supplementary file 1 [file Table_1.docx]

**TableS1.Clinical features of negative MSA/MAA associated ILD in DM patients**

|  | ILD | Without ILD | P value |
| --- | --- | --- | --- |
| All DM patients (n) | 72 | 82 | 0.03 |
| DM(n,%) | 63(87.5%) | 65(79%) |  |
| CADM(n,%) | 8(11.1%) | 7(8.5%) |  |
| JDM(n,%) | 1(1.4%) | 10(12.2%) |  |
| Age at onset (years) | 47.1±12 | 39.1±18.7 | <0.001 |
| Gender(F,%) | 48(66.7%) | 43(52.4%) | 0.07 |
| Disease duration (weeks) | 4.5(2.3,13.5) | 12(3,36) | 0.007 |
| Heliotrope sign(n,%) | 42(58.3%) | 65(79.2%) | 0.005 |
| mechanic’s hand(n,%) | 37(51.4%) | 13(15.8%) | <0.001 |
| Gottron’s sign (n,%) | 49(68%) | 43(52.4%) | 0.049 |
| muscle weakness(n,%) | 48(66.7%) | 57(69.5%) | 0.7 |
| Arthralgia(n,%) | 34(47.2%) | 20(24.3%) | 0.002 |
| Fever(n,%) | 23(31.9%) | 23(28%) | 0.59 |
| Raynaud’s phenomenon(n,%) | 6(8.3%) | 6(7.3%) | 0.8 |
| Perlungual erythematous(n,%) | 8(11.1%) | 4(4.9%) | 0.15 |
| Skin ulcer(n,%) | 7(9.7%) | 6(7.3%) | 0.59 |
| ANA positive(n,%) | 27(42.2%) | 30(41.7%) | 0.9 |
| T cells counts(cell/ul) | 925(504,1318) | 1046(590,1406) | 0.15 |
| CK(IU/L) | 79(42,287) | 80(37.7,329) | 0.7 |
| LDH(IU/L) | 252(204,367) | 241(173,330) | 0.14 |
| Ferritin(ng/ml) | 244(123,669) | 114(78,226) | 0.008 |
| CRP(mg/dl) | 0.53(0.23,0.96) | 0.24(0.14,0.66) | 0.016 |
| ESR( mm/h) | 17(7,36) | 10.5(5,24) | 0.09 |
| Elevated CEA(n,%) | 14(24.1%) | 4(5.7%) | 0.003 |
| Elevated CA7-24(n,%) | 8(30.7%) | 10(27%) | 0.7 |
| Elevated CA125(n,%) | 11(19%) | 7(9.9%) | 0.13 |
| Elevated CA199(n,%) | 10(17.5%) | 8(8.5%) | 0.3 |
| Elevated CA153(n,%) | 12(21%) | 3(4.2%) | 0.003 |
| Elevated NSE(n,%) | 12(41.4%) | 8(34.8%) | 0.62 |
| Elevated CYFRA21-1(n,%) | 18(56.3%) | 13(39.8%) | 0.17 |

*****(TableS1 Breakdown)：Continuous data were presented as M (mean) ± SEM (standard error of the mean) or medians (interquartile range).Binary data were presented as n (%) of the patients.DM: dermatomyositis; CADM:Clinical amyopathic dermatomyositis; JDM:Juvenile dermatomyositis; CK: creatine kinase; LDH: Lactate Dehydrogenase; CRP:c reactive protein ;ESR:erythrocyte sedimentation rate;

**Table S2**. Associations between the HRCT patterns and mechanic’s hand or arthralgia/arthritis

| HRCT patterns | Mechanic’s hand | | P value | Arthralgia/arthritis | | P value |
| --- | --- | --- | --- | --- | --- | --- |
|  | with | without |  | with | without |  |
| OP | 13 | 9 | 0.529 | 8 | 14 | 0.206 |
| NSIP | 24 | 25 |  | 26 | 23 |  |
| UIP | 1 | 0 |  | 1 | 0 |  |

UIP:usual interstitial pneumonia; NSIP:nonspecific interstitial pneumonia; OP:organizing pneumonia
